# Compromised Dynamic Cerebral Autoregulation in Patients with a Right-to-Left Shunt: A Potential Mechanism of Migraine and Cryptogenic Stroke

**DOI:** 10.1371/journal.pone.0104849

**Published:** 2014-08-14

**Authors:** Zhen-Ni Guo, Yingqi Xing, Jia Liu, Shuang Wang, Shuo Yan, Hang Jin, Yi Yang

**Affiliations:** 1 Neuroscience Center, Department of Neurology, the First Norman Bethune Hospital of Jilin University, Chang Chun, China; 2 Center for Neurovascular Ultrasound, the First Norman Bethune Hospital of Jilin University, Chang Chun, China; 3 Shenzhen Institutes of Advanced Technology, Chinese Academy of Sciences, Xueyuan Avenue, Shenzhen University Town, Shenzhen, China; University Medical Center Goettingen, Germany

## Abstract

**Background and Purpose:**

The relationship between right-to-left shunts (RLS) and migraine and cryptogenic stroke is not well understood. In this study, we investigated whether RLS are associated with impairment of dynamic cerebral autoregulation (dCA), which may play a role in migraine and cryptogenic stroke.

**Methods:**

Sixty-six migraineurs were enrolled in the study, including 36 non-RLS patients and 30 RLS patients. Non-invasive continuous cerebral blood flow velocity and arterial blood pressure were recorded simultaneously from each patient by using transcranial Doppler and servo-controlled plethysmograph, respectively. Transfer function analysis was applied to derive autoregulatory parameters of gain, phase difference (PD), and autoregulation index.

**Results:**

The PD in migraineurs with RLS was 50.6±22.9 degrees, which was significantly lower than that observed in the non-RLS group (67.2±18.2 degrees, P<0.001). The PD in the large RLS group (45.4±22.6 degrees) was significantly lower than that of the small RLS group (64.9±17.1 degrees, P<0.01) and non-RLS group (P<0.001); however, the PD in the small RLS group was similar to that of the non-RLS group. The PD in the permanent group (48.8±19.9 degrees) was similar to that of the latent group (52.6±26.1 degrees), and both were significantly lower than that of the non-RLS group (P<0.05). The autoregulation index results were similar to the PD findings.

**Conclusions:**

dCA is impaired in migraineurs with large RLS, and this may represent a potential mechanism linking RLS, migraine, and cryptogenic stroke.

## Introduction

A number of studies have provided substantial evidence for a strong relationship between right-to-left shunts (RLS) and migraine and cryptogenic stroke [1]–[3]. However, the mechanisms underlying this relationship are not well understood. A widely accepted theory is that subclinical metabolites and emboli from the venous system circumvent the lungs and directly enter systemic circulation (paradoxical embolism), leading to migraine and embolism [3]–[7]. This theory is not infallible though, as the cerebrovascular system can clear or wash out emboli if cerebral perfusion is well maintained. Therefore, we suspect that cerebral autoregulation, a control mechanism that maintains cerebral blood flow despite changes in arterial blood pressure, is impaired in patients with RLS. Impairment of autoregulation may lead to hypoperfusion, resulting in dysfunction related to the clearance of emboli and metabolites [8], which may eventually lead to metabolite aggregation and embolism.

It has been reported that both migraine and stroke may be associated with abnormal cerebrovascular autoregulation [9]–[12]. However, these studies did not investigate potential associations between impaired autoregulation and RLS.

To elucidate the causes of migraine and cryptogenic stroke, we investigated if, in addition to paradoxical embolisms, RLS may be associated with impairments in dynamic cerebral autoregulation (dCA), which may play a role in these diseases. In the present study, we assessed dCA in migraine patients with or without RLS and attempted to understand the relationship between RLS and dCA.

## Methods

### Participants

The study design was approved by the Ethics Committee of the First Norman Bethune Hospital of Jilin University, China. Written informed consent was obtained from all participants or parents/guardians. For minors, we explained the details, feasibility, and potentially harmful effects to the participants and their parents/guardians before the informed consent was signed. Minors had the opportunity to express their views, and we respected their views.

We conducted a prospective study of consecutive migraine patients treated at the Department of Neurology, First Hospital of Jilin University, China, from April 2013 to December 2013. Each patient was diagnosed with migraines by two neurologists according to the International Headache Society Criteria [13].

The age criterion was greater than 14 but less than 50 years old. Patients with extracranial/intracranial artery stenosis or occlusion and atrial fibrillation were excluded. The clinical workup consisted of a thorough physical examination, laboratory tests, transcranial Doppler (TCD, MultiDop X2, DWL, Sipplingen, Germany), carotid ultrasound (IU22, Phillips, Andover, Massachusetts, USA), contrast-enhanced transcranial Doppler (cTCD, MultiDop94; DWL, Sipplingen, Germany), and magnetic resonance imaging (MRI, 1.5 T, Signa, General Electric Medical Systems, Milwaukee, WI). We used T1-weighted sequences (repetition time, 450 ms; echo time, 9 ms; field of view, 240 mm; matrix, 256×224; slice thickness, 5 mm; intersection gap, 2 mm; and 15.63 kHz bandwidth) and fast spin echo T2-weighted sequences (repetition time, 4200 ms; echo time, 85 ms; echo train length of 11; field of view, 240 mm; matrix, 256×256; slice thickness, 5 mm; intersection gap, 2 mm; and 15.63 kHz bandwidth) as one index to diagnose cerebral infarction, and used T2 to measure infarct size. Each patient was diagnosed with cryptogenic stroke by two neurologists, according to comprehensive evaluations of stroke etiology, including complete medical history of vascular risk factors, clinical examinations, laboratory tests, MRI, TCD, carotid ultrasound, and electrocardiography. According to Trial of Org 10172 in Acute Stroke Treatment (TOAST) classifications, patients were excluded if they had large vessel stroke, cardiac embolism, lacunar stroke, or other definite causes of stroke.

Patients were divided into an RLS group or non-RLS group; the RLS group was divided into a small RLS group (1–10 microbubbles during normal breathing or after the Valsalva maneuver) and a large RLS group (>10 microbubbles during normal breathing or after the Valsalva maneuver). In addition, the RLS group was divided into two groups: permanent (RLS occurred at rest and after the Valsalva maneuver) and latent (RLS occurred only after the Valsalva maneuver).

### cTCD protocol

An 18-gauge needle was inserted into the cubital vein when the patient was in the supine position. Insonation of one middle cerebral artery (MCA) was performed using TCD. Contrast agent was prepared using 9 mL isotonic saline solution, 1 mL air, and a drop of the patient’s blood; this was vigorously mixed between two 10-mL syringes via a 3-way stopcock. After 30 mixing cycles, the contrast agent was injected as a rapid bolus. The first injection was performed during normal respiration (rest), and the second injection was performed 5 s prior to the start of a 10-s Valsalva maneuver. The maximum number of bubbles recorded from the MCA during normal breathing, or after the Valsalva maneuver, were taken as an estimate of the maximum degree of shunt [5].

### DCA Protocol

Baseline arterial blood pressure (ABP) was measured at the brachial artery by using an automatic blood pressure monitor (Omron 711) and continuous cerebral blood flow velocity (CBFV) was recorded using TCD in the bilateral MCA at a depth of 45–60 mm. The continuous ABP was measured non-invasively using servo-controlled plethysmograph (Finometer PRO, Netherlands) on the middle finger, and endtidal CO_2_ (EtCO_2_) was monitored using a capnograph (MultiDop X2, DWL, Sipplingen, Germany) with a face mask attached to the nasal cannula.

dCA examination was performed on the second day after the cTCD examination to ensure that patients avoided smoking, drinking, and nicotine for at least 12 h. The examination was performed in a quiet, dedicated research laboratory at a controlled temperature of 20–24°C, with external stimuli minimized. Baseline ABP was measured after 10 min in a supine position. Then, continuous CBFV and ABP were recorded simultaneously from patients in a supine position for 10 min. Recorded data were then used to assess dCA.

### Data Analysis

Recorded data were processed using a personal computer and MATLAB (a suite of commercial software for data processing). Beat-to-beat alignment of the data was achieved using a cross-correlation function to remove possible time lags. A third-order Butterworth low-pass filter (cutoff at 0.5 Hz) was then applied as an anti-alias filter before downsampling the data to 1 Hz. dCA was evaluated using transfer function analysis (TFA) [14]. The transfer function between ABP and CBFV was calculated as the quotient of the cross-spectrum of the two signals and the autospectrum of ABP in the frequency domain. Impulse and frequency responses were derived from the TFA. In the time domain, a step response was calculated by convolution between the impulse response and a normalized step to reveal how the CBFV responded to a step change in ABP. The autoregulation index (ARI) was determined from the highest coefficient correlation between the step response estimated from the recoded data and 10 pre-defined step responses from Tiecks’ model [15], where the index 0–9 indicates the state of cerebral autoregulation, from complete abolishment to very fast recovery from a step change [15]. A detailed calculation of ARI was proposed by Mahony *et al*. [16] In the frequency domain, we estimated the phase difference (PD), gain, and coherence function within a low frequency range (0.06–0.12 Hz) to evaluate cerebral autoregulation, where the derived parameters were considered most relevant to the hemodynamics [17]. We only used the autoregulatory parameters for the later statistical analysis if coherence within 0.06–0.12 Hz was >0.5.

### Statistical Analysis

Data were analyzed using the Statistical Program for Social Sciences version 17.0 (SPSS, IBM, West Grove, PA, USA). Measurements are expressed as mean ± standard deviation and were analyzed using Student’s *t*-tests. Count data are expressed as rates (percentages) and were identified using a chi-square test and Fisher’s exact test. The level of significance was set at *P*<0.05.

## Results

In total, 66 migraineurs (30.9±9.9 years; 19 male patients and 47 female patients) were enrolled in the study. There were 36 non-RLS patients (54.5%) and 30 RLS patients (45.5%). In the RLS group, 16 cases (53.3%) were permanent and 14 cases (43.6%) were latent; eight cases (26.7%) were small RLS and 22 cases (73.3%) were large RLS. According to MRIs, in the RLS group, seven cases (23.3%) were of cryptogenic stroke, four cases were corona radiata infarctions and three cases were basal ganglia infarctions; all of these cases were large RLS. In the non-RLS group, only three cases (8.3%) had cryptogenic stroke (all corona radiata infarctions). There was no significant difference between the RLS and non-RLS groups (*P*>0.05) and all infarcts were smaller than 1 cm.

In the RLS group, 17 cases (56.7%) presented with auras (flashes of light or blind spots). The attack frequency was 3.8±3.2 attacks/month. All attacks were accompanied by nausea, 22 cases experienced vertigo, and six experienced limb numbness during the attacks. Visual analogue scale ratings were 6.0±0.16. In the non-RLS group, six cases (16.7%) presented with auras (flashes of light). The attack frequency was 2.9±1.8 attacks/month; 23 cases experienced nausea with the attacks, 18 experienced vertigo, and attacks in four cases were associated with the menstrual cycle. Visual analogue scale ratings were 5.8±0.17.

We did not find any significant differences in sex, age, mean blood pressure, heart rate, or EtCO_2_ between RLS and non-RLS groups. Baseline characteristics are detailed in Table 1.

**Table 1:** Baseline characteristics.

	Non-right-to-left shunt(n = 36)	Right-to-left shunt(n = 30)
Male	11 (30.6%)	8 (26.7%)
Age (years)	29.2±10.7	33.0±8.6
Mean blood pressure (mm Hg)	82.3±10.7	83.7±12.0
Heart rate (beats/min)	69.7±7.5	70.9±8.1
Endtidal CO_2_ (mm Hg)	35.1±2.4	34.5±2.2

### Dynamic cerebral autoregulation

#### RLS vs. non-RLS group

**Figure 1:**
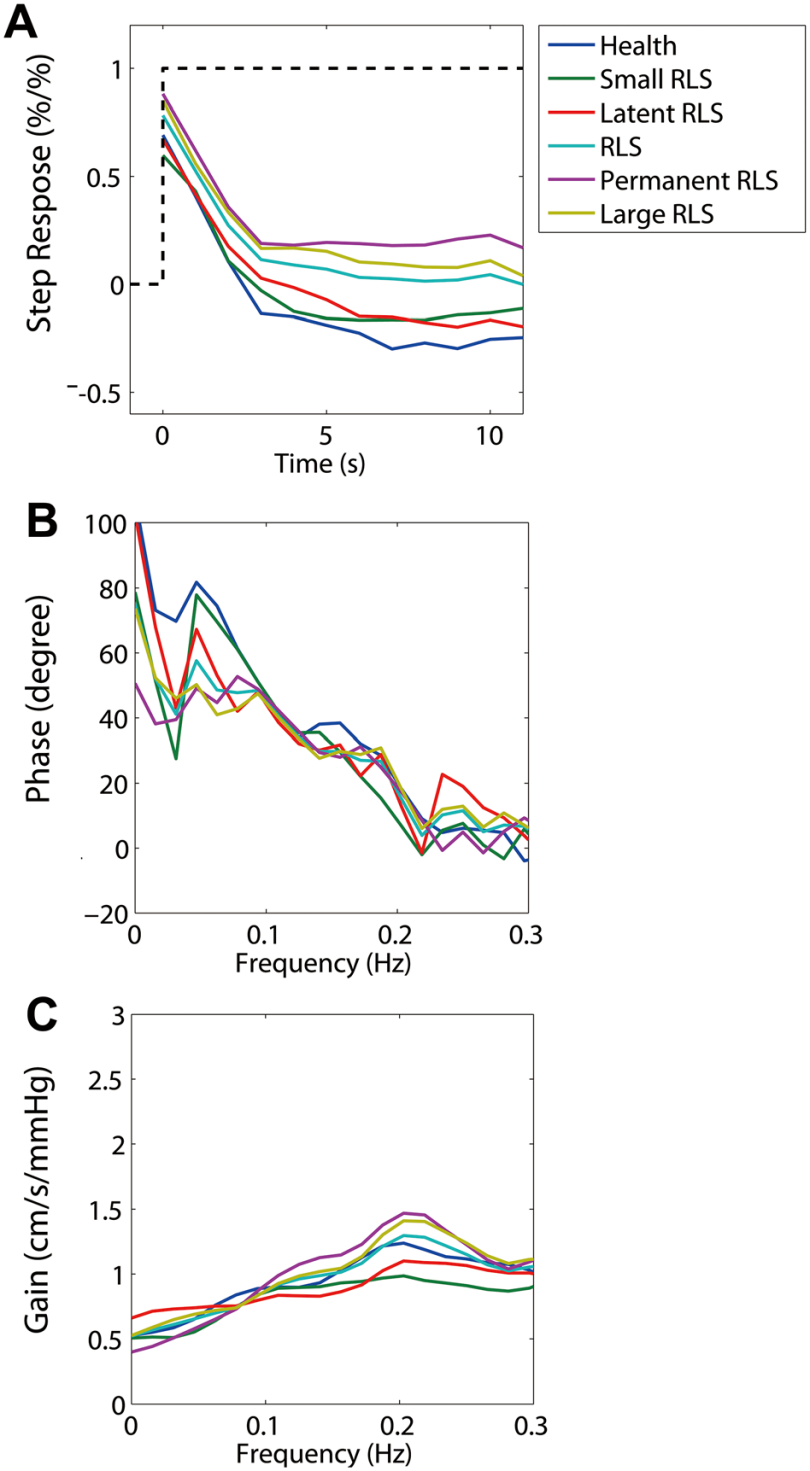
Three autoregulatory parameters derived from the transfer function. A) Step response, B) phase difference, and C) gain. Each colored line denotes an average parameter from each group. A) The dashed line is the unit step change to the transfer function, and the colored lines are responses derived from the transfer function for different groups. Both the step response and phase difference show that the best (quickest recovery from the step change and largest phase difference) dynamic cerebral autoregulation is displayed by the non-right-to-left shunt (non-RLS) group, and the worst (slowest recovery and smallest phase difference) is displayed by the large RLS group, whereas other groups are in between (statistical distribution of the parameters is shown in Figure 2).

**Figure 2:**
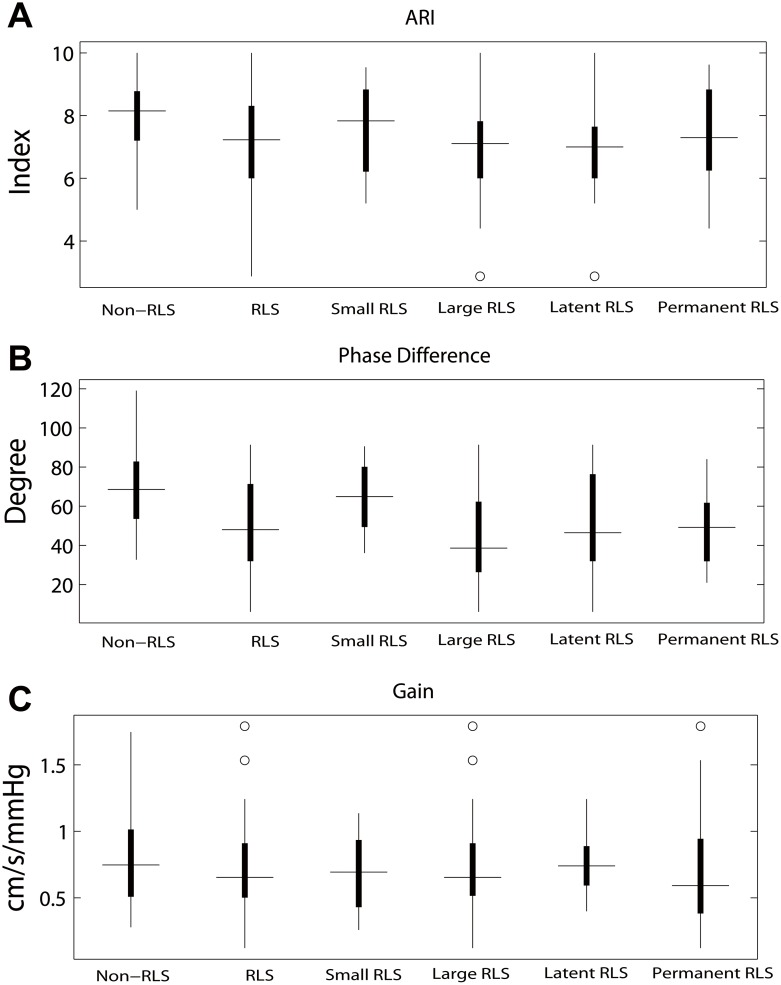
Statistical analysis of the parameters. A) The autoregulation index (ARI) from the non-RLS group is significantly higher than that of the RLS, latent RLS, and large RLS groups. B) The phase difference from the non-RLS group is significantly larger than that of the RLS, latent RLS, and large RLS groups. Moreover, the phase difference between the small RLS group and large RLS group is significant. C) There is no significant difference in gain between the groups.

The overall PD in the RLS group was significantly lower than that of the non-RLS group (t = −1.4, *P*<0.001). The overall gain in the RLS group was similar to that of the non-RLS group. The overall ARI of the RLS group was lower than that of the non-RLS group (t = −3.3, *P*<0.05, Table 2, Figures 1 and 2).

**Table 2:** Phase difference, gain, and autoregulation index (ARI) in each group.

		Phase difference(degree)	Gain(cm^−1^ s^−1^ mm Hg^−1^)	ARI
Non-RLS	Left	68.4±19.6	0.75±0.30	7.97±1.14
	Right	66.0±16.8	0.82±0.35	7.87±1.34
	Overall	67.2±18.2	0.78±0.33	7.92±1.24
RLS	Left	48.8±23.0	0.73±0.33	7.05±1.64
	Right	52.3±22.9	0.74±0.32	7.21±1.42
	Overall	50.6±22.9^a^	0.73±0.32	7.13±1.24^a^
Large RLS	Left	43.6±22.7	0.76±0.36	6.84±1.67
	Right	47.1±22.9	0.73±0.32	7.12±1.42
	Overall	45.4±22.6**^ab^**	0.74±0.34	6.98±1.54^a^
Small RLS	Left	63.2±17.9	0.64±0.26	7.63±1.49
	Right	66.6±17.2	0.75±0.32	7.46±1.46
	Overall	64.9±17.1^b^	0.69±0.29	7.54±1.43
Permanent	Left	47.0±18.7	0.72±0.43	7.28±1.70
	Right	50.7±21.5	0.69±0.36	7.29±1.56
	Overall	48.8±19.9^a^	0.71±0.39	7.29±1.61
Latent	Left	50.9±27.8	0.73±0.18	6.78±1.57
	Right	54.2±25.2	0.79±0.26	7.12±1.28
	Overall	52.6±26.1^a^	0.76±0.22	6.95±1.42^a^

RLS: right-to-left shunt.

a denotes *P*<0.05 when compared with the overall measure from the non-RLS group.

b denotes *P*<0.05 when compared with corresponding group (large RLS group vs. small RLS group and permanent group vs. latent group).

We also repeated the comparison between the RLS group and the non-RLS group after excluding patients with cryptogenic stroke. The results were similar (RLS vs. non-RLS, PD: 46.65±21.58 vs. 67.1±18.0 degrees, *P*<0.001; gain: 0.74±0.32 vs. 0.79±0.33, *P*>0.05; ARI: 7.03±1.59 vs. 7.91±1.26, *P*<0.05).

#### Large RLS vs. small RLS group

The overall PD in the large RLS group was significantly lower than that of the small RLS group (t = 3.1, *P*<0.01) and non-RLS group (t = 5.4, *P*<0.001). The overall gain in the large RLS group was similar to that of the small RLS and non-RLS groups. The overall ARI of the large RLS group was similar to that of the small RLS group but lower than that of the non-RLS group (t = 3.6, *P*<0.001, Table 2, Figures 1 and 2).

The overall PD, gain, and ARI in the small RLS group were similar to those recorded in the non-RLS group (Table 2, Figures 1 and 2).

#### Permanent vs. latent group

The overall PD in the permanent group was similar to that of the latent group but significantly lower than that of the non-RLS group (t = 4.6, *P*<0.001). The overall gain and ARI in the permanent group were similar to those in the latent and non-RLS groups (Table 2, Figures 1 and 2).

The overall PD and ARI in the latent group were lower than that of the non-RLS group (PD: t = 2.7, *P*<0.05; ARI: t = 3.4, *P*<0.05), whereas the gain was similar between the two groups (Table 2, Figures 1 and 2).

## Discussion

In our study, the autoregulatory parameters, PD and ARI, were found to be compromised in patients with RLS compared to patients without RLS, indicating a reduction in effectiveness of dCA in migraineurs with RLS. To understand the factors responsible for this, we divided the RLS group into two subgroups, using two different criteria. We found that the reduction in dCA was significant in the large RLS group compared with the small RLS group, whereas there was no difference between the permanent and latent groups. Gain was insensitive in differentiating dCA in group comparisons. These findings confirm our hypothesis and suggest that RLS is a potential cause of the impairment of dCA in migraineurs.

It has previously been reported that dCA is impaired in migraineurs [9], [18]. Müller *et al.* studied 22 migraine patients and 33 healthy controls and found that dCA phase and gain appeared to be completely different in migraineurs compared with healthy controls [9]. They suggested that this might be due to the lack of sympathetic and parasympathetic control of CBFV [9]. More recently, using a correlation coefficient index, Reinhard *et al.* found that dCA is impaired in migraine with aura; the authors suspected that this change may be associated with the increased stroke risk in migraine patients [18]. However, these studies did not investigate the possible underlying causes of the compromised dCA in migraineurs. We investigated RLS as it is a widely accepted risk factor for migraine (it is detected in more than 40% of migraineurs) and cryptogenic stroke [5]–[7], [19]. Indeed, we found evidence of dCA impairment in migraineurs with RLS. As the degree of impairment was associated with the size of the RLS (the larger RLS, the worse the dCA), we believe that there is a direct connection between RLS and the impaired dCA.

Thus, our evidence suggests that these patients suffer pathophysiological cerebrovascular changes that manifest as impairment of dCA. One possible cause is metabolite changes in cerebral arteries. Carbon dioxide, serotonin and other substances, which directly enter the arteries from the venous system, may cause cerebral artery dilation [20], leading to a reduction in effective vasodilation. Another possible mechanism may be cortical spreading depression (CSD) [2] caused by microemboli. CSD can affect cerebrovascular reactivity [21], [22], but its effect on dCA is unknown.

The coexistence of RLS (usually due to a patent foramen ovale [PFO]), migraine, and stroke has previously been reported [1], [2]. In our study, coexistence rates of migraine and cryptogenic stroke were 23.3% in the RLS group and 8.3% in the non-RLS group; though the numerical difference is obvious, there was no significant difference between the two groups, mainly due to the relatively small sample size. The mechanisms that link these diseases are unclear, and paradoxical embolization has been proposed as a possible explanation. We suggest that, in addition to paradoxical embolization, dCA impairment may also be involved, which can result in a decrease in clearance of emboli and metabolites [8], eventually leading to clinical symptoms.

Decisions to close the PFO are controversial. In our study, patients with large RLS showed significant impairment of dCA, and these findings provide evidence to support PFO closure in order to prevent recurrent embolism. However, dCA remains intact in small RLS, suggesting that PFO closure may not be required, as there is no significant cerebral hemodynamic change.

This study has a few limitations. As cTCD is a minimally invasive examination, we were unable to recruit sufficient non-migraine, RLS-positive volunteers to compare with migraineurs. The small sample size is also a limitation. In addition, some migraineurs did not have a predominant migraine side, so there was insufficient data to compare dCA values from the predominant migraine side to the contralateral side.

## Conclusions

The current study indicates that dCA is impaired in migraineurs with large RLS, and this may be a potential mechanism linking RLS, migraine and cryptogenic stroke.
